# New reference values for maximum respiratory pressures in healthy Brazilian children following guidelines recommendations: A regional study

**DOI:** 10.1371/journal.pone.0279473

**Published:** 2022-12-29

**Authors:** Ana Aline Marcelino, Guilherme Augusto Fregonezi, Maria das Graças Lira, Fernanda de Cordoba Lanza, Íllia Nadinne Dantas Lima, Vanessa Regiane Resqueti

**Affiliations:** 1 PneumoCardioVascular Lab/HUOL, Hospital Universitário Onofre Lopes, Empresa Brasileira de Serviços Hospitalares and Departamento de Fisioterapia Universidade Federal do Rio Grande do Norte, Natal, Brazil; 2 Laboratório de Inovação Tecnológica em Reabilitação, Departamento de Fisioterapia, Universidade Federal do Rio Grande do Norte, Natal, Brazil; 3 Departamento de Fisioterapia, Universidade Federal de Minas Gerais, Minas Gerais, Brazil; 4 Universidade Federal do Rio Grande do Norte/Faculdade de Ciências da Saúde do Trairi, Santa Cruz, Brazil; Universidade Federal da Bahia, BRAZIL

## Abstract

**Objective:**

To determine reference values for maximum static respiratory pressures in healthy children from a Brazilian region, following recommendations of the European Respiratory Society (ERS) and the Brazilian Society of Pneumology and Tisiology (SBPT).

**Methods:**

A cross-sectional observational study was conducted with healthy children (6 to 11 years) of both sexes. The maximum inspiratory and expiratory pressures (PImax and PEmax, respectively) were measured using a digital manometer. Each child performed a minimum of three and a maximum of five maneuvers; three acceptable and reproducible maneuvers were considered for analysis. Minimum time for each maneuver was 1.5 seconds, with a one-second plateau, and one minute of rest between them. A stepwise multiple linear regression analysis was conducted for PImax and PEmax, considering correlations between independent variables: age, weight, and sex.

**Results:**

We included 121 children (62 girls [51%]). Boys reached higher values for maximum respiratory pressures than girls. Respiratory pressures increased with age showing moderate effect sizes (PImax: *f* = 0.36; PEmax: *f* = 0.30) between the stratified age groups (6–7, 8–9, and 10–11 years). Age and sex were included in the PImax equation (PImax = 24.630 + 7.044 x age (years) + 13.161 x sex; R^2^ = 0.189). PEmax equations were built considering age for girls and weight for boys [PEmax (girls) = 55.623 + 4.698 x age (years) and PEmax (boys) = 82.617 + 0.612 x weight (kg); R^2^ = 0.068].

**Conclusions:**

This study determined new reference equations for maximal respiratory pressures in healthy Brazilian children, following ERS and SBPT recommendations.

## Introduction

Respiratory muscle strength is generally estimated using respiratory pressure measurements, muscle contraction, and changes in lung volumes and chest wall structures [1]. Respiratory pressures can be measured using voluntary maneuvers (e.g., maximum respiratory pressures, sniff nasal inspiratory pressure), or involuntary contractions in response to phrenic nerve stimulation [1, 2].

Maximum inspiratory (PImax) and expiratory pressures (PEmax) are non-invasive and straightforward tests commonly used in clinical practice. These measurements are essential in diagnosing respiratory muscles weakness [3]. In neuromuscular diseases (e.g., amyotrophic lateral sclerosis, myotonic dystrophy, and myasthenia gravis), PImax, PEmax, sniff nasal inspiratory pressure, and spirometry values are sensitive indicators of disease severity in the earliest stages [4, 5].

Reference values for respiratory pressures vary among studies and may be related to biological characteristics of the participants (i.e., age, sex, weight, height, cultural differences), technique and equipment used, evaluator expertise, participants motivation during the test, methodological procedures, and data analysis [2, 6, 7].

Studies investigated reference values for PImax and PEmax for healthy Brazilian children [8, 9]. However, there is no consensus on the reference values reported by the studies. This may be because each study used a different protocol, equipment (e.g., aneroid manometer) [10], age groups [9, 11], and was developed in different country regions.

A lack of assessment standardization led to variations in reference values reported by each study. This variation can lead to the risk of underestimating or overestimating pressure values, with a bias to the correct diagnosis of respiratory disorders and, consequently, delay in starting the required therapy. With new studies that assess reference values, using recommended methodologies, and following methodological rigor, we can support predict the severity of dysfunctions and begin treatment within a suitable therapeutic time frame.

Therefore, this study aimed to establish new reference values for maximum static respiratory pressures in healthy children from a Brazilian region, considering European Respiratory Society (ERS), and Brazilian Society of Pneumology and Tisiology (SBPT) [1, 7, 12] guidelines. This study also aimed to compare the results, obtained from a digital manometer, with predicted values and reference equations for respiratory pressures in children reported by previous studies.

## Methods

### Ethics statement

This study followed the Declaration of Helsinki and was approved by the research ethics committee of the Onofre Lopes University Hospital under number 2.051.325. Children and parents received information about the study and signed informed assent and consent forms, respectively.

### Study design

A cross-sectional observational study was conducted in Natal/Rio Grande do Norte, Brazil. Healthy children ranging from ages 6 to 11 years old were recruited in private and public schools. Sample size was previously calculated in the study that determined reference values of sniff nasal inspiratory pressure (SNIP) [13]. Spirometry was previously developed to certify eligibility for lung function (FVC and FEV_1_> 80% of predicted and FEV_1_/FVC > 70%) [14]. This group was stratified by sex and age (6–7, 8–9, and 10–11 years old). We included children: (1) with no previous respiratory, cardiac, neurovascular, and neuromuscular diseases; (2) who no had influenza during or one week before the evaluation; (3) with no regular use of allergy medications, corticosteroids, or central nervous system depressants; (4) with no previous surgeries requiring incision in the thoracic or abdominal cavities; and (5) who had the ability to follow instructions or verbal command [12, 14, 15]. The lower age limit was established due to the risk of error in the reproducibility of the maneuvers, compromising data quality.

**Fig 1:**
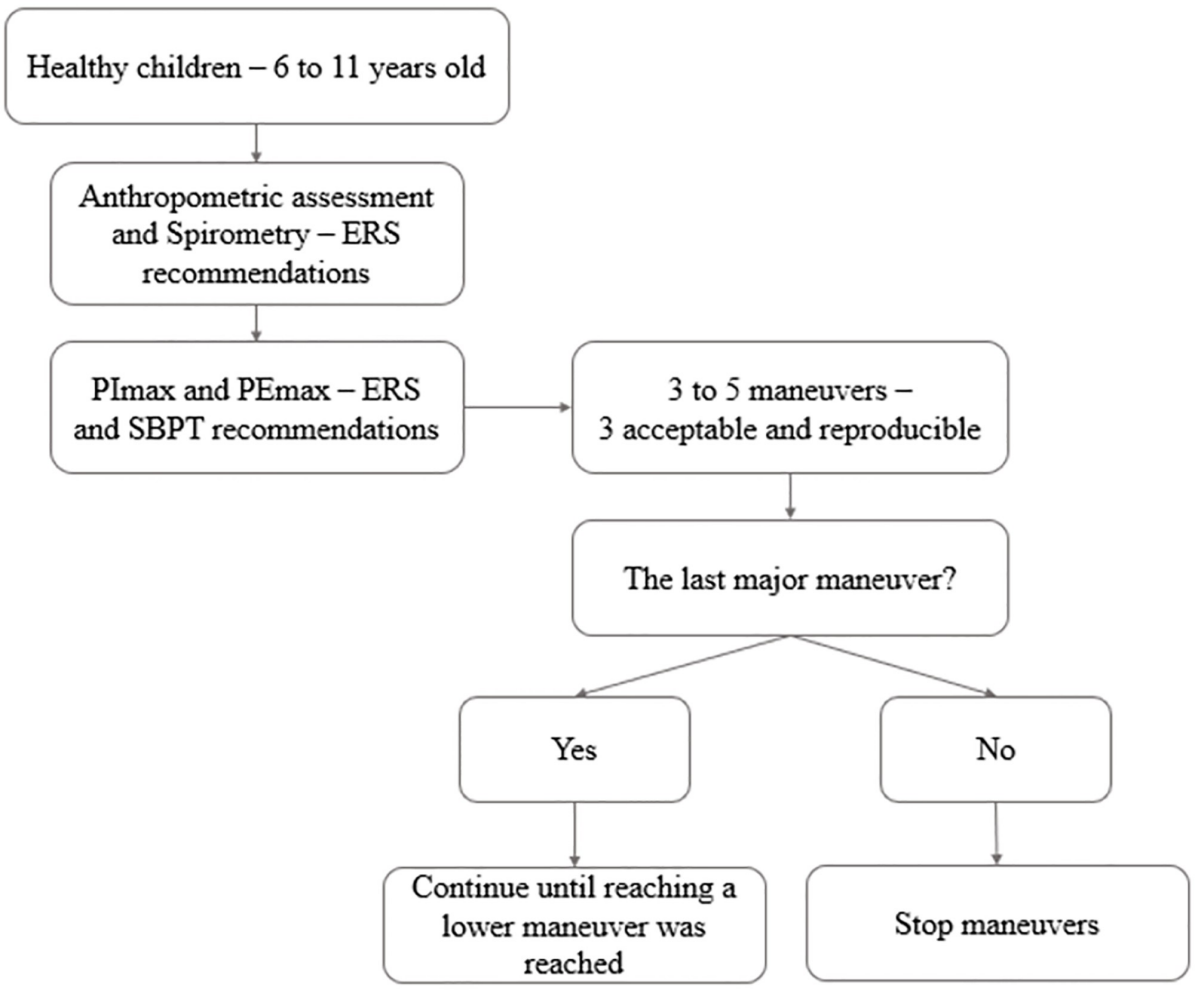
Study design flowchart.

Initially, weight and height was assessed using a mechanical scale coupled to a stadiometer (Model 110-CH, Welmy, Brazil). The percentile for body mass index was calculated using WHO Anthro Plus^®^ v3.2.2 software (World Health Organization, Switzerland). Spirometry was performed using a calibrated Koko spirometer (nSpire Health Inc, Longmont, CO, USA), with children in sitting position, as recommended by ERS [14]. Spirometry data were interpreted according to the values predicted by Mallozi published by Pereira [16]. PImax and PEmax were obtained using a digital manometer (NEPEB-LabCare/UFMG, Brazil). Data were processed by Manovac software (version 4.1), which provides mean, peak, and plateau pressure values. Procedures followed recommendations of the ERS [1] and SBPT [12], attempting to minimize errors and differences due to methodological variations between previous studies. Tests were explained, demonstrated, and conducted by the same evaluator. Children were in sitting position, with feet and trunk supported, and wearing nose clips. A disposable cylindrical mouthpiece with a leakage escape of approximately 2 mm was used to prevent glottic closure at PImax, and to minimize pressures generated by orofacial muscles and air leak during PEmax. The evaluator held hands to children’s cheeks during maneuvers to prevent air leakage. PImax was performed after a maximum expiration (i.e., close to residual volume). PEmax was performed after a maximum inspiration (i.e., close to total lung capacity). Minimum time for each maneuver was 1.5 seconds with one second plateau, and one minute of rest between each maneuver. Each child performed a minimum of three and a maximum of five maneuvers to achieve three acceptable (without air leak and adequate duration) and reproducible maneuvers (values of two largest maneuvers could not differ more than 10%, and value of the third largest maneuver could not differ more than 20% from the highest value) [7]. Last maneuver could not be the greatest since it would indicate learning effect. When this occurred, evaluation should continue until a lower value was reached [7]. Fig 1 summarizes the procedures performed. The highest value of the maximum mean pressure was used in data analysis.

### Data analysis

Data were analyzed using SPSS 22.0 (IBM Corp., Chicago, IL, USA). Kolmogorov-Smirnov test was used to evaluate data distribution; all data were found to be normally distributed. Descriptive data were presented as mean ± standard deviation. Unpaired t-tests were used to compare PImax and PEmax between sexes. One-way ANOVA with Bonferroni’s post hoc adjustment was used to compare respiratory pressures between three age groups. Effect sizes were calculated using Cohen’s *f* for subgroup analysis, classifying as small (< 0.25), moderate (0.25–0.40), and large (> 0.40) [17]. For intergroup analysis, Cohen’s *d* was used, classifying as small (< 0.5), moderate (0.5–0.8), and large (> 0.8) [17]. A stepwise multiple linear regression analysis was performed for PImax and PEmax considering correlations with independent variables (age, weight, and sex). This analysis adds the independent variables to the model and from a significant p-value, we can determine the best model that can predict our dependent variable. Variables that best correlated and made the regression model better explain the variation of the dependent variable were added to the model. In addition, we compared PImax and PEmax values found in this study with respiratory pressures and reference equations determined by previous studies, by obtaining the mean difference between values. Significance level of all tests was set at *p* < 0.05, and 95% confidence interval was considered. T-test was used based on the mean and standard deviation (SD) of previous values found in the Stefanutti and Fitting study. A power of 99% and α = 0.05 revealed the need for a total of 120 children [18].

## Results

One hundred twenty seven children were assessed, of whom 6 were excluded due to inability to perform the respiratory pressure maneuvers. Therefore, 121 children comprised the final sample, with 62 girls. Anthropometric characteristics and pulmonary function are described in Table 1.

**Table 1:** Anthropometric characteristics and pulmonary function stratified by sex.

Variables	Total group (n = 121)	Girls (n = 62)	Boys (n = 59)
	*Mean ± SD*	*Mean ± SD*	*Mean ± SD*
Age (years)	8.41 ± 1.49	8.32 ± 1.42	8.50 ± 1.58
Weight (kg)	34.18 ± 10.18	33.44 ± 9.23	34.96 ± 11.12
Height (cm)	133.60 ± 9.68	133.50 ± 9.40	133.60 ± 10.05
BMI percentile	70.87 ± 29.29	70.62 ± 27.20	71.12 ± 31.58
FVC (L)	1.95 ± 0.45	1.87 ± 0.41	2.05 ± 0.48
FVC (%)	105.10 ± 12.58	103.90 ± 13.08	106.40 ± 11.99
FEV_1_ (L)	1.72 ± 0.34	1.67 ± 0.31	1.77 ± 0.36
FEV_1_ (%)	100.10 ± 10.56	100.60 ± 11.62	99.65 ± 9.39
FEV_1_/FVC	88.73 ± 6.28	90.17 ± 5.70	87.21 ± 6.55

BMI: body mass index; FVC: forced vital capacity; FEV_1_: forced expiratory volume in one second; FVC (%) and FEV_1_ (%): percentage predicted.

Table 2 presents the variation of maximum respiratory pressures between sexes and age groups. PImax differed significantly between sexes with a moderate effect size; however, no differences and a small effect size were found for PEmax. There were significant differences between age groups for PImax and PEmax. The two oldest group (8–9 and 10–11 years) presented higher respiratory muscle strength in comparison with children aged 6–7 years.

**Table 2:** Differences in maximum respiratory pressures stratified by sexes and age groups.

**Maximum Respiratory Pressures**	**Girls**		**Boys**		**p**	**Cohen’s *d* (ES)**
	*Mean ± SD*		*Mean ± SD*			
PImax (cmH2O)	83.26 ± 27.17		97.73 ± 30.09		0.006*	0.50
PEmax (cmH2O)	94.73 ± 25.70		104.00 ± 26.11		0.051	0.37
	**6–7 years (n = 39)**	**8–9 years (n = 45)**		**10–11 years (n = 37)**	**p**	**Cohen’s *f* (ES)**
	*Mean ± SD*	*Mean ± SD*		*Mean ± SD*		
PImax (cmH2O)	77.44 ± 27.25	91.91 ± 25.63		101.9 ± 31.26	< 0.001*	0.36
PEmax (cmH2O)	89.10 ± 22.61	103.1 ± 28.19		105.3 ± 24.72	0.011*	0.30

Unpaired t-test was performed for comparisons between sexes; One-way ANOVA for comparisons between age groups;

*p < 0.05; Cohen’s *d* and *f* (ES): effect sizes.

Table 3 presents regression models generated for each maximum respiratory pressure. Independent variables height, weight, age, and sex were positively correlated with PImax, but only age and sex were maintained in the equation. PEmax was positively correlated with height, weight, and age; the variable age was maintained in the equation for girls and weight for boys. Reference equations and lower limits (LL) are described below:


$$\text{PImax}=24.630+7.044\;\text{x}\;\text{age}\;\left(\text{years}\right)+13.161\;\text{x}\;\text{sex},\;\text{for}\;\text{sex}:\;0\;\text{for}\;\text{girls}\;\text{and}\;1\;\text{for}\;\text{boys};\;\text{LL}=\text{predicted}\;\text{value}–43.93$$

$$\text{PEmax}\;\left(\text{girls}\right)=55.623+4.698\;\text{x}\;\text{age}\;\left(\text{years}\right);\;\text{LL}=\text{predicted}\;\text{value}–41.16$$

$$\text{PEmax}\;\left(\text{boys}\right)=82.617+0.612\;\text{x}\;\text{weight}\;\left(\text{kg}\right);\;\text{LL}=\text{predicted}\;\text{value}–41.81$$

**Table 3:** Prediction equations for PImax and PEmax in both sexes.

	Model	Β	SE (β)	95% CI (β)	R^2^
PImax	Constant	24.630	13.994	3.081; 52.342	0.189
	Age	7.004	1.631	3.814; 10.275	
	Sex	13.161	4.869	3.519; 22.803	
PEmax (girls)	Constant	55.623	19.000	17.617; 93.629	0.068
	Age	4.698	2.251	0.196; 9.201	
PEmax (boys)	Constant	82.617	11.008	60.574; 104.659	
	Weight	0.612	0.300	0.010; 1.213	0.068

β: regression coefficients; SE (β): standard error of the regression coefficients; CI 95% (β): 95% confidence interval of the regression coefficients; R^2^: coefficient of determination.

**Fig 2:**
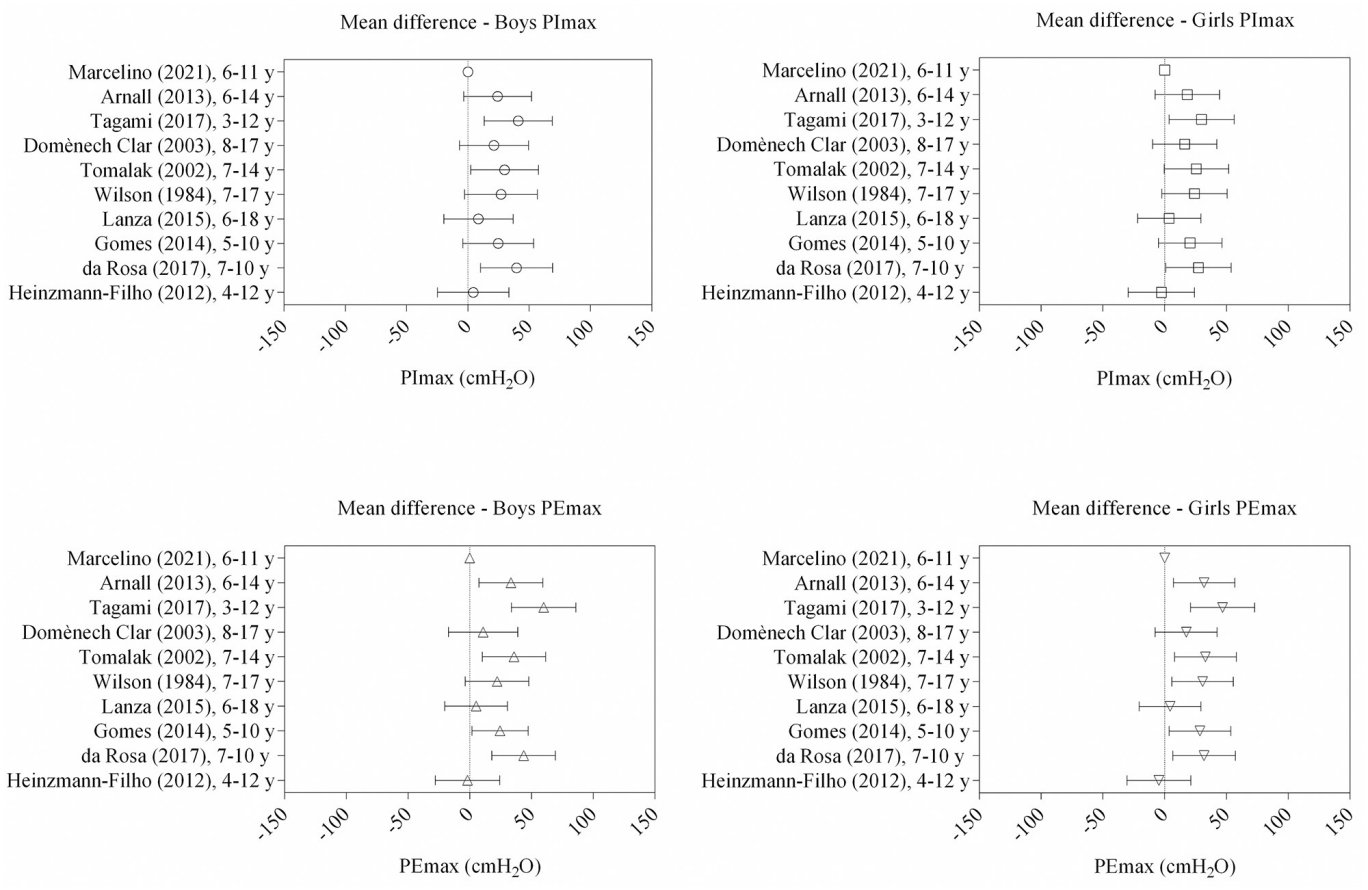
Mean difference between maximum respiratory and expiratory pressures (PImax and PEmax, respectively) found in the present and previous studies, according to sex; y = years.

We calculated the mean difference between maximum respiratory pressures obtained in this study with reference values from previous studies according to sex (Fig 2). These results are similar to the results reported by Heinzmann-Filho et al. and Lanza et al., which also developed reference equations with Brazilian children [8, 10]. Table 4 shows the main methodological characteristics of previous studies.

**Table 4:** Methodological characteristics of previous studies.

Studies	N	Country	Age	Type of manometer	Single or multicenter	Independent variables	R^2^ variation
Marcelino, 2021	121	Brazil	6–11	Digital	Single	Age, sex, and weight	0.07–0.19
Arnal, 2013	534	USA	6–14	Aneroid	Single	Age	0.08–0.26
Tagami, 2017	218	Japan	3–12	Digital	Single	Age, weight, and height	0.13–0.22
Domènech-Clar, 2003	392	Spain	8–17	Digital	Single	Age, weight, and height	0.21–0.51
Tomalak, 2002	296	Poland	7–14	Digital	Single	Age	0.09–0.18
Wilson, 1984	235	England	7–17	Aneroid	Single	Age, weight, and height	0.05–0.37
Lanza, 2015	450	Brazil	6–18	Aneroid	Multicenter	Age, sex, and BMI	0.31–0.34
Gomes, 2014	148	Brazil	5–10	Aneroid	Single	Age, BMI, and height	0.25–0.63
Da Rosa, 2017	399	Brazil	7–10	Digital	Single	Age, weight, and height	0.14–0.22
Heinzmann-Filho, 2012	171	Brazil	4–12	Digital	Single	Age and weight	0.46–0.59

BMI: body mass index; R^2^: coefficient of determination.

**Fig 3:**
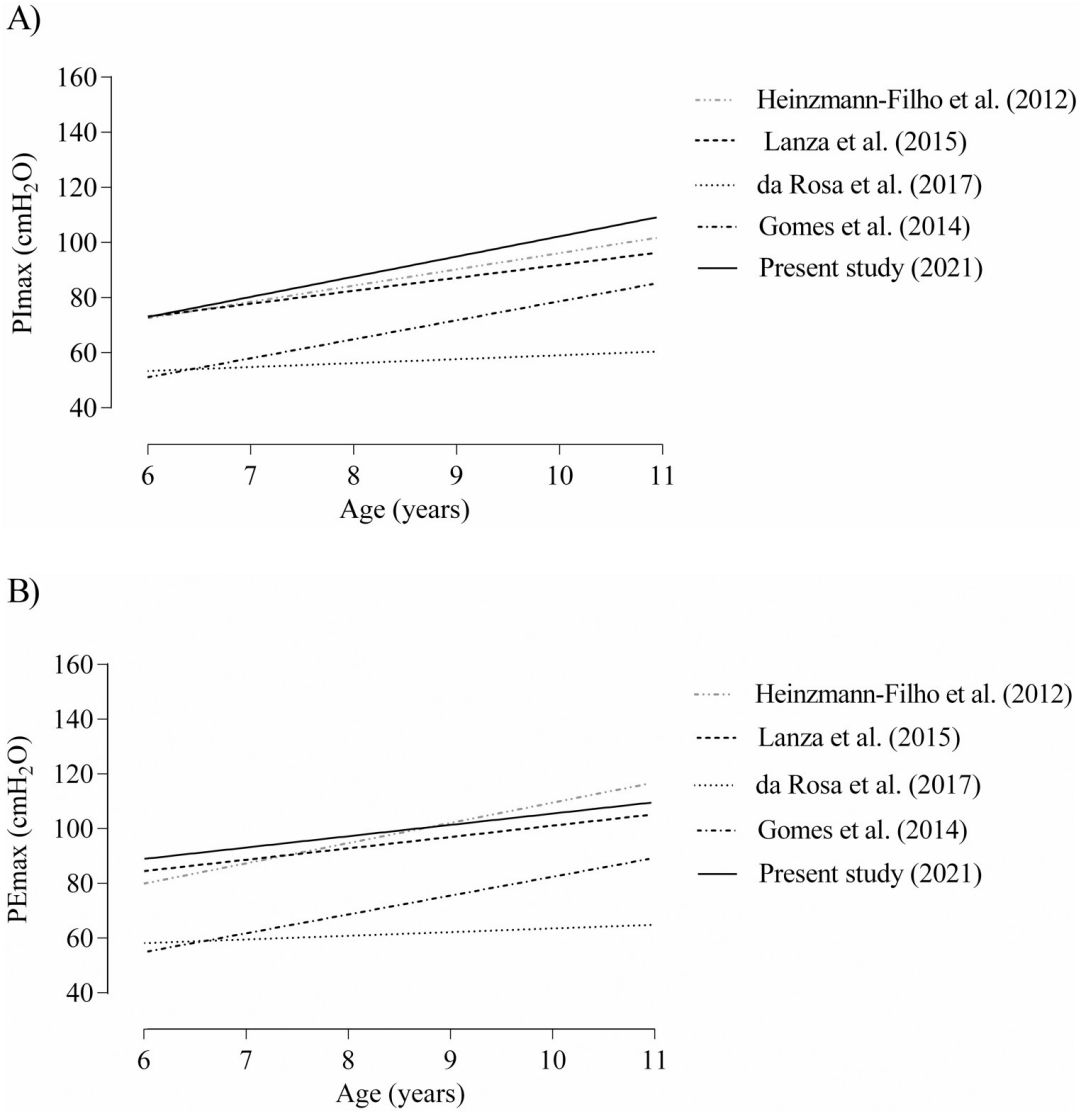
Trend lines of predicted values for PImax (A) and PEmax (B) observed in the present study and in previous Brazilian studies, according to age groups.

Estimated PImax and PEmax mean values obtained in the present study were also compared with previous Brazilian studies, according to age groups. Prediction lines are drawn in Fig 3A and 3B.

## Discussion

We determined new reference values for maximal respiratory pressures in healthy Brazilian children between 6 and 11 years. Previously, several studies [8, 9, 19, 20] indicated reference values for maximal respiratory pressures in healthy children. However, they had limitations such as methodological, and individual variations inherent to the study sample.

Pessoa et al., (2014) [7] also followed the recommendations from the ERS and SBPT. They reported that maneuvers methodological standardization and use of digital equipment with software influence maximum mean pressure values (mean maximum pressure sustained for one second) in adults. However, we observed a lack of information regarding maneuvers and equipment, including the software used to choose the best measure in reference values for children. Therefore, it is unspecified whether the interpretation was performed only visually, or some mathematical model of software was incorporated. Graphical visual analysis without mathematical interpretation implies the chance of misinterpretation, leading to an incorrect diagnosis.

This study used a digital device with software to establish reference values for maximum respiratory pressures in healthy children, as recommended by the ERS and SBPT. In this study, only boys had PImax values significantly higher than girls, despite reaching higher mean values in both respiratory pressures. Boys reached 14% higher PImax and 10% higher PEmax than girls. Similar results were reported by Heinzmann-Filho et al. (2012) [8] and Lanza et al. (2015) [10], registering a difference between sexes of 9% for PImax and 10% for PEmax, and 8% for PImax and 10% for PEmax, respectively. According to previous studies [2], sexual hormonal differences and greater muscle mass in boy [10] explain the differences between sexes, or even differences in respiratory mechanics, since boys can be more elongated.

Respiratory pressures increased with age, and moderate effect sizes were found between groups (6–7, 8–9, and 10–11 years). This increase seems to be linear for both respiratory pressures, but more evident for PImax. Besides differences between age of children included in the studies, we observed a linear relation in most analyzed studies [8, 10, 11], except for da Rosa et al. (2017) [9], suggesting that age is an important independent variable for reference equations.

Comparing with the present study, most previous studies showed a positive mean difference in maximum pressures with higher respiratory pressures in this sample than in previous studies [9, 11, 19, 20]. The findings were more similar to those reported by Heinzmann-Filho et al. (2012) [8] and Lanza et al. (2015) [10]. Some factors still lead to variations between studies results, namely cultural and regional differences, methodological dissimilarities, types of instruments, and participants’ motivation. Heinzmann-Filho et al. (2012) [8] allowed children to place their hands on their cheeks to avoid increasing oral pressure during PEmax. This aspect may lead to variations due to difference in pressure applied by each participant. Different from the recommendation for digital manometers [3], Lanza et al. (2015) [10] used an aneroid manometer, which can under or overestimate pressures measurements.

Age and sex were the best predictors for PImax, age was the best predictor for PEmax in boys, and weight for PEmax in girls. Studies indicate age as the variable that best composes regression models [8, 9, 19, 20], followed by weight [8, 9, 20–22], and height [9, 11, 21, 22]. Some studies even included body mass index [10, 11] in their models. We reached coefficients of determination (R^2^) of 0.189 for PImax and 0.068 for PEmax. Heinzmann-Filho et al. (2012) [8] and Gomes et al. (2014) [11] obtained R^2^ close to or greater than 0.5 for both respiratory pressures. Tomalak et al. (2002) [22] reached R^2^ values similar to this study for Polish children (0.177 and 0.098 for PImax in boys and girls, respectively, and 0.170 and 0.091 for PEmax). Although all studies included age and anthropometric measurements in equations, they showed large variation in R^2^ values. Notably, other independent variables could influence respiratory pressures and change the results (i.e., thorax size, diaphragmatic circumference, and hormonal level).

Although some studies followed the recommendation of using digital manometers, the software used to select the largest maneuvers, peak, plateau, and mean pressure was not informed [8, 9]. Some studies that used aneroid manometer [10, 11] also showed this limitation of not describing how maneuver choice was carried out. These distinctions between studies methodologies may explain differences in respiratory pressures. This study has the advantage of using a digital manometer developed in Brazil (NEPEB, Minas Gerais), with its software for choosing reproducible and acceptable maneuvers that show peak, plateau, and mean pressure values. Although we followed a complete methodology for performing maneuvers using a suitable manometer, it has some limitations. We neither include children from other Brazilian regions nor added evaluations that could interfere with results, such as thorax size and diaphragmatic circumference.

In conclusion, this study determined new reference equations for maximum respiratory pressures in healthy children, including variables such as age, sex, and weight, and using a methodology recommended by ERS and SBPT. Thus, we reinforce the importance of using the recommended methodology, adequate equipment to avoid over or underestimating measures, and to sufficiently report how the maneuvers were selected.
